# Noise-induced vocal plasticity in urban white-crowned sparrows does not involve adjustment of trill performance components

**DOI:** 10.1038/s41598-018-36276-5

**Published:** 2019-02-13

**Authors:** Katherine E. Gentry, David A. Luther

**Affiliations:** 1 George Mason University Biology Department, 4400 University Dr., Fairfax, 22030 USA; 2 Present Address: Purdue University Department of Biological Sciences, Lilly Hall of Life Science, 915W. State St., West Lafayette, IN 47906 USA

## Abstract

Background noise can interfere with acoustic communication and subsequently influence signaling behavior. Immediate signaling flexibility (ISF) is a context-dependent form of behavioral plasticity that allows animals to temporarily change their acoustic behavior in response to noise fluctuations and potentially improve the chances of successful communication in noisy environments. The adaptive value of ISF is ultimately contingent on the response of the intended receiver, and there are differential effects on receiver response depending on which signal component is modified. However, there is scant research on whether ISF involves modification of a signal component specifically linked to mate attraction or territory defense. Our study addresses this knowledge gap and provides important insight into whether males employ short-term signal modification in a manner that could affect mate pairing success in birds. Specifically, we explore the maladaptive potential of ISF in the San Francisco, California population of *Zonotrichia leucophrys nuttalli* by testing for changes in trill bandwidth and rate—the specific trill structure components known to influence the receiver’s perception of vocal performance in this species—before and during noise broadcast experiments. Although *Zonotrichia leucophrys nuttalli* are capable of ISF, we found no evidence that noise induces temporary adjustment of the trill structure traits used by receivers to assess vocal performance.

## Introduction

Background noise can interfere with acoustic communication and subsequently influence signaling behavior and the evolution of acoustic communication^1^. Efforts to improve signal detection and discrimination in the presence of background noise can lead to changes in acoustic behavior through a mechanism of selection, such as cultural transmission, an involuntary change in vocal effort (e.g. Lombard effect)^2^, or a decisive action taken by the signaler (e.g. adjustment in timing of vocalization)^3–5^. A context dependent form of behavioral plasticity, known as immediate signaling flexibility (ISF), allows animals to temporarily change their acoustic behavior in response to fluctuations in background noise and can involve adjustments of the amplitude; spectral or temporal features of acoustic signals; or the timing of signaling behavior^6,7^. However, the manner and extent to which the signal is modified varies both across and within species depending on the species’ developmental plasticity, microevolutionary responses, or capacity for behavioral flexibility^8^, individual flexibility and prior experience of the signaler^9,10^, as well as the perception of noise^5,11^.

Temporary modification to the spectral features of an acoustic signal is an especially well documented form of noise-induced ISF. Some birds, for example, raise the minimum frequency of their songs during bouts of relatively loud, low-frequency background noise (e.g. *Ammodramus bairdii*^12^, *Contopus virens*^13^, *Haemorhous mexicanus*^14^, *Emberiza schoeniclus*^15^, *Phylloscopus collybita*^16^, *Erithacus rubecula*^17^, *Zosterops lateralis*^18^, *Poecile atricapillus*^10^). Other species decrease the maximum frequency of their song in response to short-term high amplitude noise exposure (e.g. *Zonotricia leucophrys nutalli*)^19^. An increase in minimum frequency or decrease in maximum frequency reduces signal bandwidth, which can improve signal detection if it concentrates signal frequencies within a more sensitive hearing range of the receiver^20–22^. Alteration of the spectral structure of acoustic signals can also reduce spectral overlap between the signal and the background noise, improving the likelihood of signal detection and discrimination by intended receivers^1,22^ (but see^23^).

Although ISF can improve the chances of successful communication in noisy environments, its adaptive value is ultimately contingent on the response of the receiver. In songbirds, the link between signal modification and receiver response is explained by the fact that (1) song functions in mate attraction and territory defense and (2) song is used by receivers to assess signaler quality^24,25^. Indeed, songs with a smaller bandwidth and/or increased minimum frequency (common forms of noise-induced signal modification) are known to elicit relatively weak responses, in comparison to other local songs with greater bandwidth, from territorial rivals^26–29^. It is important to note, however, that not all components of the song, or song types convey the same information^30–33^; thus, there are differential effects on receiver response depending on which song component is modified.

For example, in many species whose song includes a trill component, the trill structure—including trill bandwidth and trill rate— is used by receivers to assess signaler quality (vocal performance reviewed in^34^). Until there is sufficient evidence that ISF occurs specifically in a song component that influences a signalers’ ability to defend his territory or attract mates, we can only continue to speculate about the effect that ISF has on vocal performance. Addressing this knowledge gap will provide important insight into whether short-term signal modification has implications for territory defense or mate pairing success.

**Figure 1:**
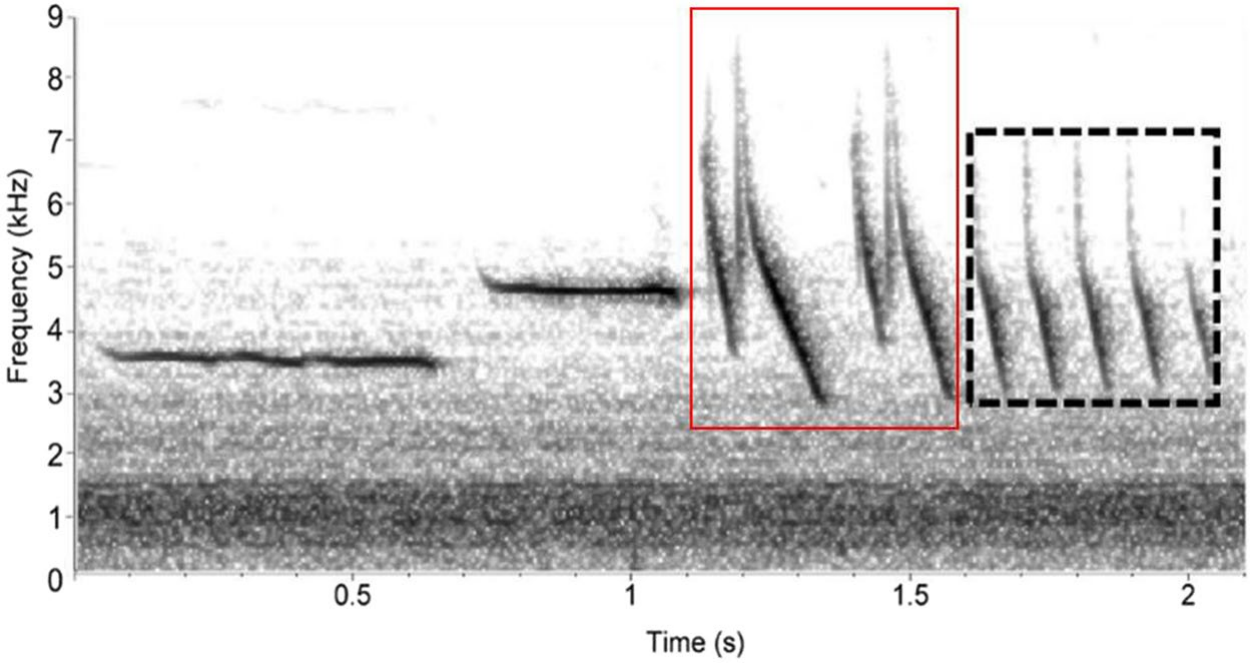
Spectrogram image of NWCS song recorded during the low frequency noise broadcast. The different components of the song are identified within the text and the terminal trill structure is outlined in a dashed black line. We did not observe ISF in the trill structure; however, minimum frequency of the complex note structure (outlined in red solid line) has increased over time with urban background noise levels^44^.

Here we use the Nuttall’s white-crowned sparrow (*Zonotrichia leucophrys nuttalli*; hereafter, NWCS), a model system for acoustic behavior modification, to explore the maladaptive potential of ISF. The NWCS sings a single stereotyped song type, comprised of introductory, complex note, and terminal trill components (Fig. 1), and experimental evidence confirms: (1) both trill bandwidth and trill rate are important during male-to-male territorial interactions, (2) male receivers use each trill trait when assessing vocal performance of competitors, and (3) the strength of receivers’ response is not influenced by the background noise level of their territory^27,35,36^. There is also evidence of spectral ISF in the urban population of NWCS of San Francisco, CA^19^, but further research is needed to elucidate if ISF in NWCS involves the adjustment of the terminal trill structure such that it would affect vocal performance. As with other species whose song consists of a trill component^37–40^, NWCS that sing at a high trill rate and/or wide bandwidth elicit responses in receivers that suggest they are perceived as higher quality males^35^. Thus, ISF could negatively affect the receiver’s perception of vocal performance if it also involves signal modification that reduces trill bandwidth or trill rate.

## Results

There was no significant difference in terminal trill bandwidth during the experimental noise broadcast relative to that measured prior to the broadcast (F_1,15.1_ = 3.33, P = 0.09; pre-noise: LMS ± SE = 2676.02 ± 155.88; during noise: LMS ± SE = 2546.30 ± 155.88), nor was there a significant difference in the terminal trill rates (F_1,15.0_ = 0.26, P = 0.62; pre-noise: LMS ± SE = 13.37 ± 1.07; during noise: LMS ± SE = 13.18 ± 1.07). See Table 1 for a summary of the song measurements per study site. In addition, bandwidth and trill measurements did not change with background noise levels (bandwidth: F_1,12.1_ = 0.07, P = 0.79; trill rate: F_1,8.5_ = 3.16, P = 0.11). Lastly, trill bandwidth and trill rate did not change with recording distance from speaker (F_1,12.95_ = 0.003, P = 0.96; F_1.10.1_ = 0.13, P = 0.73, respectively). See Supplementary Material 1 for full model summary output.

**Table 1:** Summary of song measurements.

Pre-noise Broadcast		
Study Site	Trill Rate (s) (µ ± SD)	Trill Bandwidth (Hz) (µ ± SD)
BATE-W	14.28 ± 1.33	2747.91 ± 349.73
GOGA	8.75 ± NA	3639.10 ± NA
INPO	13.30 ± 0.04	2428.96 ± 85.25
LAEN	11.24 ± 1.46	2618.61 ± 407.30
LAME	16.25 ± NA	2390.23 ± NA
LODUBABE	14.77 ± 3.28	2558.61 ± 1045.19
**During Noise Broadcast**		
BATE-W	14.09 ± 1.75	2744.79 ± 302.81
GOGA	9.00 ± NA	3466.85 ± NA
INPO	12.87 ± 0.64	2378.49 ± 153.62
LAEN	11.24 ± 1.84	2383.39 ± 268.89
LAME	16.50 ± NA	2153.36 ± NA
LODUBABE	14.06 ± 1.62	2255.63 ± 266.47

## Discussion

We found that urban NWCS did not adjust trill bandwidth or trill rate in response to experimental, low frequency noise broadcasts. Previous research on the same urban population of NWCS demonstrated that males are capable of ISF, as they temporarily increased song amplitude and reduced the overall maximum frequency and bandwidth of their song in response to increased noise levels^19,41^. A reduction in bandwidth and increase in signal amplitude is thought to improve signal detection in noise^20,42^; thus, our results suggest that ISF may facilitate communication in noisy conditions without affecting trill performance components in our species.

Our results are particularly informative because of the extensive amount of research available on the acoustic behavior of the NWCS and how it changes in relation to background noise. For instance, a higher minimum frequency song structure has evolved over time through cultural transmission within the same urban NWCS population^43,44^. The increase in minimum frequency is substantial enough to enhance the signal-to-noise ratio in urban locations where the background noise levels are high enough for the critical ratio to be relevant for minimum song frequency^45^. NWCS also exhibit ISF amplitude adjustments; specifically, they increase song amplitude when background noise is higher^41^. Similarly, experimental noise broadcasts confirmed urban NWCS temporarily decrease maximum frequency, and thus bandwidth, of songs during high levels of background noise^19^. The concomitant enhancement of signal-to-noise ratio^45^, decrease in bandwidth^19,43,44^, and increase in amplitude^41^ should significantly improve the chances of song detection and discrimination in the presence of relatively high levels of background noise^20^. From these studies, we can infer that the adaptive value of ISF is potentially augmented in especially noisy locations, as a song with a higher minimum frequency means that song bandwidth is comparatively smaller when the male temporarily reduces maximum frequency in response to increased noise levels. Still, transmission experiments are needed to determine the extent to which ISF specifically improves signal detection and active space of NWCS songs; the results from which will allow for a more precise assessment of the adaptive value of ISF in a noisy urban soundscape.

Similarly, more playback experiments are required to determine the precise threshold at which signal adjustment begins to influence the perception of trill performance in receivers. This threshold is likely species-specific, as it is in part dependent on the receivers’ ability to detect signal adjustments. While we did not find that noise-induced ISF involves adjustment of trill bandwidth and trill rate at respective effect sizes of 200 Hz and 1.0 Hz (or greater), it is unlikely that smaller effect sizes would have biological relevance as Henry and Lucas^46^ show that auditory filters for white-crowned sparrows (measured per equivalent rectangular bandwidth) widen as frequency increases (e.g. ~500 Hz at 2000 Hz to ~700 Hz at 4000 Hz). Given that a male’s trill structure in our urban population has an average minimum frequency of 3229 Hz and an average maximum frequency of 4000 Hz (averages calculated from Phillips and Derryberry 2017 dataset; N = 101 urban males from the same study population as ours), it is more likely that only large spectral adjustments to the trill structure are detectable and have the potential to alter the perception of vocal performance. In fact, NWCS respond more strongly in experimental playbacks to high performance songs with an average trill rate of 11.8 Hz and trill bandwidth of 4144.6 Hz than low performance songs with an average trill rate of 6.3 Hz and trill bandwidth of 3104.8 Hz (Phillips and Derryberry 2017). In contrast, the broader auditory filters allow for greater temporal resolution of rapid frequency modulation and amplitude modulation, likely making relatively small adjustments in trill structure noticeable to receivers^46^. Nevertheless, our finding that NWCS do not appear to significantly adjust their trill rate or bandwidth in response to experimental noise broadcasts support the notion that maintenance of trill structure is important for mate attraction and territory defense in our species. This conclusion aligns with the results derived from larger sample sizes that indicated trill structure and performance was more consistent within males than between males (N = 51 rural NWCS and N = 109 urban NWCS)^35^.

It is important to note, however, that males with relatively low vocal performance occupy noisier territories^27,47^, so consistency in vocal performance suggests that a spatial relationship also exists between background noise levels and the perceived quality of male territory-owners. Existence of a pattern between noise levels and perception of male quality is further supported by the finding that vocal activity, another potential indicator of male quality, decreases across a territory noise gradient within the same urban NWCS population^48^. It remains to be seen if a spatial relationship also exists between background noise levels and NWCS reproductive success, such a relationship is conceivable given that noise causes glucocorticoid-signaling dysfunction and induces stress and hypocorticism in both nestlings and adult birds^49^. In fact, experimental evidence shows reduced baseline cortisol levels in white-crowned sparrow (*Zonotrichia leucophrys*) chicks exposed to noise broadcasts during their first five days of life^50^. Besides the hormonal symptoms of chronic stress, reduced hatching success of bird species is also reported in relatively noisy environments^49^.

Our experimental design allowed us to detect ISF of song structure^19^ and assess if the components of the song associated with vocal performance were affected by ISF, which they were not. We modeled the experimental design after similar broadcast noise experiments on Great Tits (*Parus major*) by Halfwerk and Slabbekorn^28^ that involved recording songs and syllable types of songs before, during, and after broadcast noise. However, it was difficult to obtain a large sample size of males that would sing before, during, and after the broadcast noise; many birds flew away and/or stopped singing when noise was presented and did not necessarily sing again or continue to sing after the broadcast ended. For these reasons, we were unable to obtain enough song recordings post-broadcast to test whether trill structure differed between three time treatments; thus, our results should be interpreted with caution. Still, a comparison between songs recorded prior and during the noise broadcast is arguably more valuable than a comparison between songs recorded during and after the noise broadcast, as it excludes the possibility of a noise carry-over effect on singing behavior potentially biasing our interpretation of the results.

While we observed that ISF of song structure did not seem to affect the signal components that are used for assessing vocal performance in our species, this may not be the case in other species. The effect of ISF on the perception of signaler quality by receivers is understudied, despite widespread literature on the topic of noise-induced ISF. Although there is a large and still growing literature on noise-induced changes in songs (reviewed in^5^), some of which focus on ISF, only two, to our knowledge, have specifically focused on ISF and examined a song trait that was *a priori* experimentally confirmed to influence the outcome of male-male contests and influence female mate choice^10^: the consistency of the inter-note frequency ratio in the song of the black-capped chickadee (*Poecile atricapillus*)^10^ and the duration of songs of house finches (*Haemorhous mexicanus*)^51^. While ISF occurred in the form of song shifting in the chickadees, the inter-note frequency ratio did not change. This finding is similar to ours in that it does not suggest that noise-induced ISF negatively affects vocal performance. However, the results are not as widely applicable across all bird species; as far as we know, few other species use relative consistency in frequency ratios between notes to convey information about social dominance^52,53^. In the house finches ISF occurred with the finches shortening the duration of their songs in the presence of experimentally broadcast urban noise, whereas Nolan and Hill^33^ showed that females prefer longer songs. In the case of the house finches ISF adjustments negatively impacted the quality of the song.

In contrast, other studies on noise-induced ISF offer less insight into the potential effect on mate attraction and territory defense. For instance, signal complexity in European robins (*Erithacus rubecula*) temporarily changes during relatively high amplitude noise^17^, but as far as we know the function of signal complexity in this species is linked to sex differentiation and does not correlate with sexual selection or reproductive success^54^. Another study observed that male reed buntings (*Emberiza schoeniclus*) had a correlation between the ISF of a songs’ minimum frequency and reduced breeding success in noisier^15^ territories. However, the role of minimum frequency in mate attraction and territory defense for reed buntings was unknown.

Presently, our understanding of how NWCS receivers respond to noise-induced signal adjustment is limited to amplitude and the trill components tested within this population^35,36,55^. Therefore, to more fully understand the adaptive value of ISF, more research is needed to clarify whether other noise-induced signal adjustments in other song components, such as the complex note, are important for sexual selection, and whether these adjustments affect perception of vocal performance and change receiver response as well. It is also unknown whether NWCS exhibit ISF in the form of short-term temporal avoidance, like the white throated sparrows (*Zonotrichia albicolis*) that wait to sing until a car has passed and background noise returns to lower amplitudes^56^. Song consistency, including consistent timing among songs within bouts, is an additional indicator of vocal performance in other songbird species^57,58^. Therefore, it is possible that noise-induced disruption to the consistent expression of inter-song intervals could reduce the receivers’ perception of NWCS male quality. Research on this matter is another important next step to improving our understanding of the adaptive value of ISF in NWCS. In regard to the adaptive value of noise-induced ISF in other species, however, we recommend future research (1) test for ISF in signal components known to influence reproductive success, and (2) involve a study species for which the function of their signal components is known.

## Methods

### Experimental noise broadcasts

**Figure 2:**
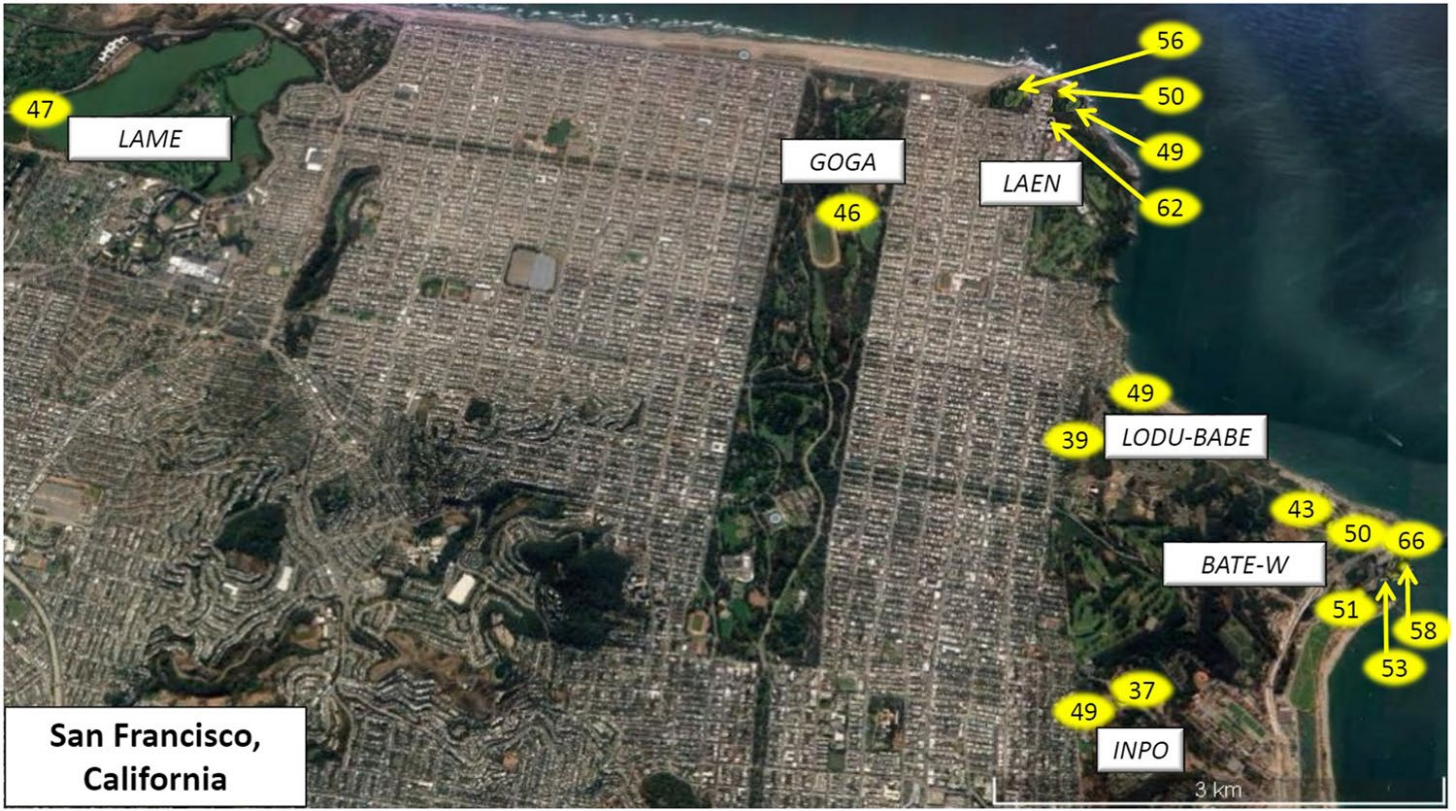
Study site map of experimental locations scattered throughout San Francisco, California. Satellite image extract was exported from the Google Earth Pro® (https://www.google.com/earth/desktop/); map data: Google, Landsat/Copernicus. Study site labels are included. In addition, a yellow, oval-shaped symbol is placed over each approximate location where a noise broadcast experiment was conducted; otherwise the symbol is attached to a yellow arrow that points to the approximate experimental location. Territory noise levels (L_Aeq_) taken at the time of the experiment are also noted within the symbols.

Following the experimental procedure detailed in Gentry *et al*.^19^, we conducted noise broadcast experiments on 16 free-living urban NWCS males across six study sites throughout San Francisco, California (Fig. 2) between 0500–1100 h from April 10^th^ through May 18^th^ of 2016 (during the NCWS breeding season, post-territory establishment). We used the four-minute sound file of Halfwerk and Slabbekoorn^28^ for the low-frequency noise broadcast, the type of noise typical of anthropogenic activity in the city (and previously confirmed as the dominate source of abiotic noise in our urban sites)^59^. For each male, we recorded songs prior to the experimental noise and during the experimental noise. However, we did not record songs if there were abnormal noise sources present (e.g. jackhammer or other construction sounds; planes or drones flying overhead; parade/marathon participants passing through; rain or high wind; fog horns; etc.).

The experimental noise level was set to 78 L_Aeq_ at one meter from the speaker using a calibrated sound level meter (SLM; Larson Davis model 831, firmware version 2.206 with the settings of A frequency weighting, Fast time weighting, linear averaging, and 0.0 dB gain) that calculated 1 s L_Aeq_, or A-weighted equivalent continuous level value, based on the sound pressure levels sampled 10 times a second. The amplitude of the noise broadcast was then gradually increased from 0–78 L_Aeq_ over twenty seconds.

The noise broadcast experiment commenced when at least two pre-broadcast songs were recorded with a Sennheiser ME66 directional microphone and a Marantz PMD660 digital recorder (Marantz America, Inc., Westbury, NY, USA) and the male was singing within 5–17 m of the speaker (Peavey Solo Portable PA System Battery amplifier connected to an IPod Nano). The Marantz PMD660 recordings were flat-weighted and low-cut filtered (40–20,000 Hz), and were recorded in wav file format at a sampling rate of 44.1 kHz and saved to internal memory as 24 bit. To maintain stable signal intensity at the point of the receiver, we redirected the speaker toward the male if he changed song post. We made voice annotations to note any changes in the male’s distance from the speaker, and we made sure to never lose sight of him during the recording period that immediately preceded the experimental noise broadcast as well as during the four-minute experimental noise broadcast. An experiment was considered successful when at least two songs were recorded before and during the experimental noise. Any songs recorded after the experimental noise were not measured, since the majority of individuals either flew away before the four minutes of noise ended or did not continue to sing through the entire broadcast or after it ended.

It is unlikely that we rerecorded the same male twice, because in all but one case, test subjects were separated by at least 120 m from one another with several other NWCS territories established between them. Two experiments were conducted 75 m apart, but in that instance, one of the test subjects was individually identifiable as he was previously marked with color bands. The National Park Service IACUC approved this research (all methods were performed in accordance with protocol 0427‐R). The U.S. Fish and Wildlife Service (MB679782‐1), the Bird Banding Laboratory (23900), and the California National Resources Agency (SC‐6799) granted permission for this research. Permission for research in the urban sites was granted by the Golden Gate National Recreation Area (SCI‐0007), the San Francisco Parks and Recreation (041415).

After each noise experiment, a calibrated sound level meter (see above) was used to collect sound pressure levels (SPL). The post-broadcast background noise was measured over sixty seconds with the Larson Davis oriented upwards, at approximately 1.5 meters high. Sixty second A-weighted equivalent continuous level values (L_Aeq_) were calculated using the 1 s L_Aeq_ values by taking the mean of the pressures and converting back to the dB scale. Males were sampled across territories that spanned a broad spectrum of background noise (Table 2; Fig. 2), specifically background noise levels ranged 37–66 L_Aeq_ (µ ± SE = 51.4 ± 1.3 L_Aeq_). All reported sound pressure levels are in units of L_Aeq_ re 20 µPa (8–20 kHz).

**Table 2:** Mean territory noise level at all study sites in San Francisco, CA.

Study site	Mean Territory Noise Level (L_Aeq_) (µ ± SD)	N territories
BATE-W	55.2 ± 6.8	6
GOGA	51.0 ± NA	1
INPO	41.5 ± 4.0	2
LAEN	54.0 ± 0.8	4
LAME	54.0 ± NA	1
LODUBABE	43.5 ± 7.5	2

### Song analysis

We measured songs that we could clearly discern from the background noise in the spectrogram viewing window. For instance, if a car drove by and we happened to record the bird singing over the car noise, we would not measure that particular song; instead, we would measure songs recorded prior to the car passing or recorded after the car passed and the noise subsided. We measured the terminal trill structure using the sound analysis software program Raven v1.5^60,61^. We measured at least two songs before and at least two songs during noise for each male. Terminal trill measurements included peak frequency contour (PFC) bandwidth (Hz; a measure of the difference in frequency range between PFC maximum and minimum frequencies (the highest and lowest frequency traced by the PFC tool, respectively)). We reduced subjectivity of spectral measurement by using the PFC tool, as it traces signals according to the points of peak energy along the contour. We also measured trill rate (Hz), calculated by dividing the 90% duration measure (s; length of the vocalization which contains 90% of the energy) of the trill by the number of terminal trill notes^59^. More details on sound measurements are given in Gentry *et al*.^19^.

### Statistical analyses

All statistical analyses were done using R statistical software^62^. For each test subject, terminal trill measurements were averaged for songs recorded immediately prior to the experimental noise as well as for the songs recorded during the experimental low-frequency noise broadcast. The ‘qqp’ function in packages ‘car’ and ‘MASS’ was used to confirm our data were normally distributed^63,64^. To determine if individuals adjusted their terminal trill structure in immediate response to changes in low frequency noise level at the time of experimental noise, we built two linear mixed models using ‘lmer’ in the R package ‘lmerTest’^65^ and tested for plasticity of each terminal trill measure (PFC bandwidth and trill rate). In addition to the within-subject categorical fixed effect, ‘time’ (pre-noise or during noise broadcast), we included the between-subject fixed effects, ‘distance’ from the broadcast speaker (m) and territory background noise (‘LAeq’), to account for variation in signal-to-noise ratio. We chose an additive model structure after confirming that model fit was not improved by a three-way interaction term or any two-way interaction terms (AIC increased with addition of interaction term; F-test of model structures containing interaction terms indicate all interactions are nonsignificant (see Supplementary Material 1 for F-test results output). We included ‘test subject’ as a random intercept to account for the two repeated measures (pre-noise and during noise measures). Prior to fitting our models, we also confirmed through likelihood ratio tests that goodness of fit was improved by a hierarchical random effect structure that also included ‘study site’ (P < 0.05). In addition, we ran a post hoc power analysis test for each of our linear mixed models using SimR, and confirmed our experimental design had sufficient statistical power to detect biologically revelant effect sizes for the ‘time’ variable^66^: At our sample size of 16, there is sufficient power to detect a bandwidth effect size as small as 200 Hz (80.7% power) and trill rate effect size as small as 1.0 note/sec (77.7% power) (see Supplementary Material 1 for power analysis results). We report P values using a Satterthwaite approximation for the degrees of freedom. We used the doBy package^67^ to compute least squares means for ‘time’ using adjusted degrees of freedom. The datasets generated during and analyzed during the current study are available from the corresponding author on reasonable request.

## Electronic supplementary material

Supplementary Information
