# Supplementary material for: Noise-induced vocal plasticity in urban white-crowned sparrows does not involve adjustment of trill performance components
Source: Sci Rep. 2019 Feb 13;9:1905. doi: 10.1038/s41598-018-36276-5 (PMC6374513; doi:10.1038/s41598-018-36276-5)
Supplement: Supplementary file 1 — Supplementary Information [file 41598_2018_36276_MOESM1_ESM.pdf]

Title: Noise-induced vocal plasticity in urban white-crowned sparrows does not involve adjustment of trill performance components

Katherine E. Gentry and David A. Luther

Appendix 1

BANDWIDTH MODEL SUMMARY:

Estimate for fixed effect equates to effect size and is highlighted

---

```
> modela <- lmer(meanPFCbw~time+distance+LAeq+(1|test subject)+(1|study site)
, data=meanISFfull)
> summary(modela)
Linear mixed model fit by REML. t-tests use Satterthwaite's method ['lmerModL
merTest']
Formula: meanPFCbw ~ time+distance+LAeq+(1|test subject)+(1|study site)
   Data: meanISFfull

REML criterion at convergence: 424.2

Scaled residuals:
     Min       1Q   Median       3Q      Max
-1.62527 -0.41768  0.05255  0.33687  1.97265

Random effects:
 Groups   Name        Variance Std.Dev.
 Test subject (Intercept) 132896   364.5
 Study site   (Intercept)  75754   275.2
 Residual              40481   201.2
Number of obs: 32, groups:  Test subject, 16; Study site, 6

Fixed effects:
            Estimate Std. Error       df t value Pr(>|t|)
(Intercept) 3092.240   1224.721    7.083   2.525   0.0392 *
timeprepb    129.726     71.135   15.000   1.824   0.0882 .
distance      -2.903     42.994    8.755  -0.068   0.9477
LAeq         -10.162     19.048    9.627  -0.534   0.6058
---
Signif. codes:  0 '***' 0.001 '**' 0.01 '*' 0.05 '.' 0.1 ' ' 1

Correlation of Fixed Effects:
          (Intr) tmprpb distnc
timeprepb -0.029
distance  -0.718  0.000
LAeq      -0.946  0.000  0.485
```

POWER ANALYSIS FOR EFFECT SIZE of 200 Hz FOR BANDWIDTH MODEL

```
> powerSim(modela1)
Power for predictor 'time', (95% confidence interval):=======================
==============================================================|
      80.70% (78.11, 83.10)

Test: Likelihood ratio
Based on 1000 simulations, (0 warnings, 0 errors)
alpha = 0.05, nrow = 32
```

TRILL RATE MODEL SUMMARY:

Estimate for fixed effect equates to effect size and is highlighted

```
> modelx <- lmer(meantrillrate90~time+distance+LAeq+(1|test subject)+(1|study
site), data=meanISFfull)
> summary(modelx)
Linear mixed model fit by REML. t-tests use Satterthwaite's method ['lmerModL
merTest']
Formula: meantrillrate90 ~ time+distance+LAeq+(1|test subject)+(1|study site)
   Data: meanISFfull

REML criterion at convergence: 124.9

Scaled residuals:
    Min      1Q  Median      3Q     Max
-1.8771 -0.4568 -0.0687  0.2909  2.0276

Random effects:
 Groups    Name             Variance Std.Dev.
 Test subject   (Intercept) 1.487    1.219
 Study site     (Intercept) 5.487    2.342
 Residual                1.041    1.020
Number of obs: 32, groups:  Test subject, 16; Study site, 6

Fixed effects:
             Estimate Std. Error        df t value Pr(>|t|)
(Intercept)  8.638234   4.714227  9.092805   1.832   0.0998
Timepre      0.183006   0.360696 15.000000   0.507   0.6193
distance    -0.009236   0.169375  9.321525  -0.055   0.9577
LAeq         0.088287   0.074572  8.797147   1.184   0.2674
---

Correlation of Fixed Effects:
          (Intr) tmprpb distnc
timeprepb -0.038
distance  -0.642  0.000
LAeq      -0.919  0.000  0.370
```

POWER ANALYSIS FOR EFFECT SIZE of 1.0 FOR TRILL RATE MODEL

```
> fixef(modelx)["timepre"] <- 1
> powerSim(modelx)
Power for predictor 'time', (95% confidence interval):=======================
==============================================================|
      77.70% (74.99, 80.25)

Test: Likelihood ratio
Based on 1000 simulations, (0 warnings, 0 errors)
alpha = 0.05, nrow = 32
```

RESULTS FOR TRILL BANDWIDTH MODEL STRUCTURES WITH INTERACTION TERMS

(ALL NONSIGNFICANT, P > 0.05).

```
lmer(meanPFCbw~time*distance*LAeq+(1|test subject)+(1|study site),meanISFful)

lmer(meanPFCbw~time+distance*LAeq+(1|test subject)+(1|study site),meanISFful)

lmer(meanPFCbw~time*distance+LAeq+(1|test subject)+(1|study site),meanISFful)

lmer(meanPFCbw~time*LAeq+distance+(1|test subject)+(1|study site),meanISFful)
```

```
> anova(time*distance*LAeq model)
Type III Analysis of Variance Table with Satterthwaite's method
                   Sum Sq Mean Sq NumDF   DenDF F value Pr(>F)
time                 3453    3453     1 12.0000  0.0871 0.7729
distance            74388   74388     1  8.4979  1.8771 0.2058
LAeq                29014   29014     1  6.4791  0.7321 0.4227
time:distance        3103    3103     1 12.0000  0.0783 0.7844
time:LAeq            2486    2486     1 12.0000  0.0627 0.8065
distance:LAeq       80353   80353     1  8.0228  2.0276 0.1922
time:distance:LAeq 3522    3522     1 12.0000  0.0889 0.7707
```

```
> anova(distance*LAeq model)
Type III Analysis of Variance Table with Satterthwaite's method
             Sum Sq Mean Sq NumDF   DenDF F value  Pr(>F)
time         134630  134630     1 15.0000  3.3258 0.08819
distance      75986   75986     1  8.4979  1.8771 0.20577
LAeq          29637   29637     1  6.4791  0.7321 0.42271
distance:LAeq 82080   82080     1  8.0228  2.0276 0.19217
```

```
> anova(time*distance model)
Type III Analysis of Variance Table with Satterthwaite's method
              Sum Sq Mean Sq NumDF   DenDF F value Pr(>F)
time            5920    5920     1 14.0000  0.1463 0.7078
distance         184     184     1  8.7552  0.0046 0.9477
LAeq           11514   11514     1  9.6266  0.2846 0.6058
time:distance  40903   40903     1 14.0000  1.0112 0.3317
```

```
> anova(time*LAeq model)
Type III Analysis of Variance Table with Satterthwaite's method
         Sum Sq Mean Sq NumDF   DenDF F value  Pr(>F)
time     163508  163508     1 14.0000  4.7754 0.04636
LAeq       9746    9746     1  9.6266  0.2846 0.60577
distance    156     156     1  8.7552  0.0046 0.94769
time:LAeq 127857  127857     1 14.0000  3.7342 0.07380
```

RESULTS FOR TRILL RATE MODEL STRUCTURES WITH INTERACTION TERMS

(ALL NONSIGNFICANT, P > 0.05).

```
Models with interaction terms:

lmer(meantrillrate90 ~ time*distance*LAeq+(1|test subject)+(1|study site),
meanISFfull)

lmer(meantrillrate90 ~ time+distance*LAeq+(1|test subject)+(1|study site),
meanISFfull)

lmer(meantrillrate90 ~ time*distance+LAeq+(1|test subject)+(1|study site),
meanISFfull)

lmer(meantrillrate90 ~ time*LAeq+distance+(1|test subject)+(1|study site),
meanISFfull)

> anova(time*distance*LAeq model)
Type III Analysis of Variance Table with Satterthwaite's method
                    Sum Sq Mean Sq NumDF   DenDF F value Pr(>F)
time               0.46458 0.46458     1 12.0000  0.4023 0.5378
distance           0.43082 0.43082     1  9.2271  0.3731 0.5561
LAeq               1.15957 1.15957     1  7.1457  1.0042 0.3490
time:distance      0.86125 0.86125     1 12.0000  0.7459 0.4047
time:LAeq          0.39864 0.39864     1 12.0000  0.3452 0.5677
distance:LAeq      0.47308 0.47308     1  8.7607  0.4097 0.5385
time:distance:LAeq 0.79057 0.79057     1 12.0000  0.6847 0.4241

> anova(distance*LAeq model)
Type III Analysis of Variance Table with Satterthwaite's method
               Sum Sq Mean Sq NumDF   DenDF F value Pr(>F)
time          0.26793 0.26793     1 15.0000  0.2574 0.6193
distance      0.38832 0.38832     1  9.2271  0.3731 0.5561
LAeq          1.04520 1.04520     1  7.1457  1.0042 0.3490
distance:LAeq 0.42642 0.42642     1  8.7607  0.4097 0.5385

> anova(time*distance model)
Type III Analysis of Variance Table with Satterthwaite's method
               Sum Sq Mean Sq NumDF   DenDF F value Pr(>F)
time          0.86384 0.86384     1 14.0000  0.8084 0.3838
distance      0.00318 0.00318     1  9.3215  0.0030 0.9577
LAeq          1.49776 1.49776     1  8.7971  1.4017 0.2674
time:distance 0.65260 0.65260     1 14.0000  0.6107 0.4475
```

```
> anova(time*LAeq model)
Type III Analysis of Variance Table with Satterthwaite's method
          Sum Sq Mean Sq NumDF   DenDF F value Pr(>F)
time     0.72869 0.72869     1 14.0000  0.6920 0.4194
Laeq     1.47592 1.47592     1  8.7971  1.4017 0.2674
distance 0.00313 0.00313     1  9.3215  0.0030 0.9577
time:LAeq  0.87073 0.87073   1 14.0000  0.8269 0.3785
```
